# Supplementary material for: Vertical Scanning Interferometry for Label-Free Detection of Peptide-Antibody Interactions
Source: High Throughput. 2019 Mar 27;8(2):7. doi: 10.3390/ht8020007 (PMC6631817; doi:10.3390/ht8020007)
Supplement: Supplementary file 1 [file high-throughput-08-00007-s001.pdf]

high-throughput

Supplementary Materials

Vertical scanning interferometry for label-free detection of peptide-antibody interactions

Andrea Palermo[1], Richard Thelen[1], Laura K. Weber[1]*, Tobias Foertsch[1], Simone Rentschler[1], Verena Hackert[1], Julia Syurik[1], Alexander Nesterov-Mueller[1]*

[1] Institute of Microstructure Technology, Karlsruhe Institute of Technology (KIT), Germany
* Correspondence: Laura.Weber@kit.edu; Alexander.Nesterov-Mueller@kit.edu

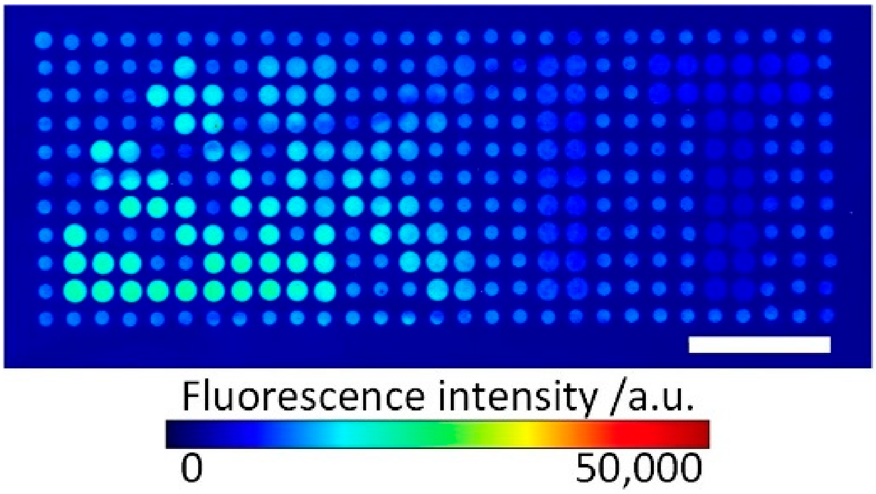

Figure S1. Overlay of the fluorescence scans of the sub array depicted in figure 3, containing the pattern of 11 x 29 spots incubated with serum (1:50), secondary antibody (labelled with AF647) at 13.3 nM and anti-HA antibody DL550 at 13.3 fM. Scans were conducted at a resolution of 2 µm/pixel, 635 nm: gain 10, 532 nm: gain 10. Scale bar represents 500 µm.

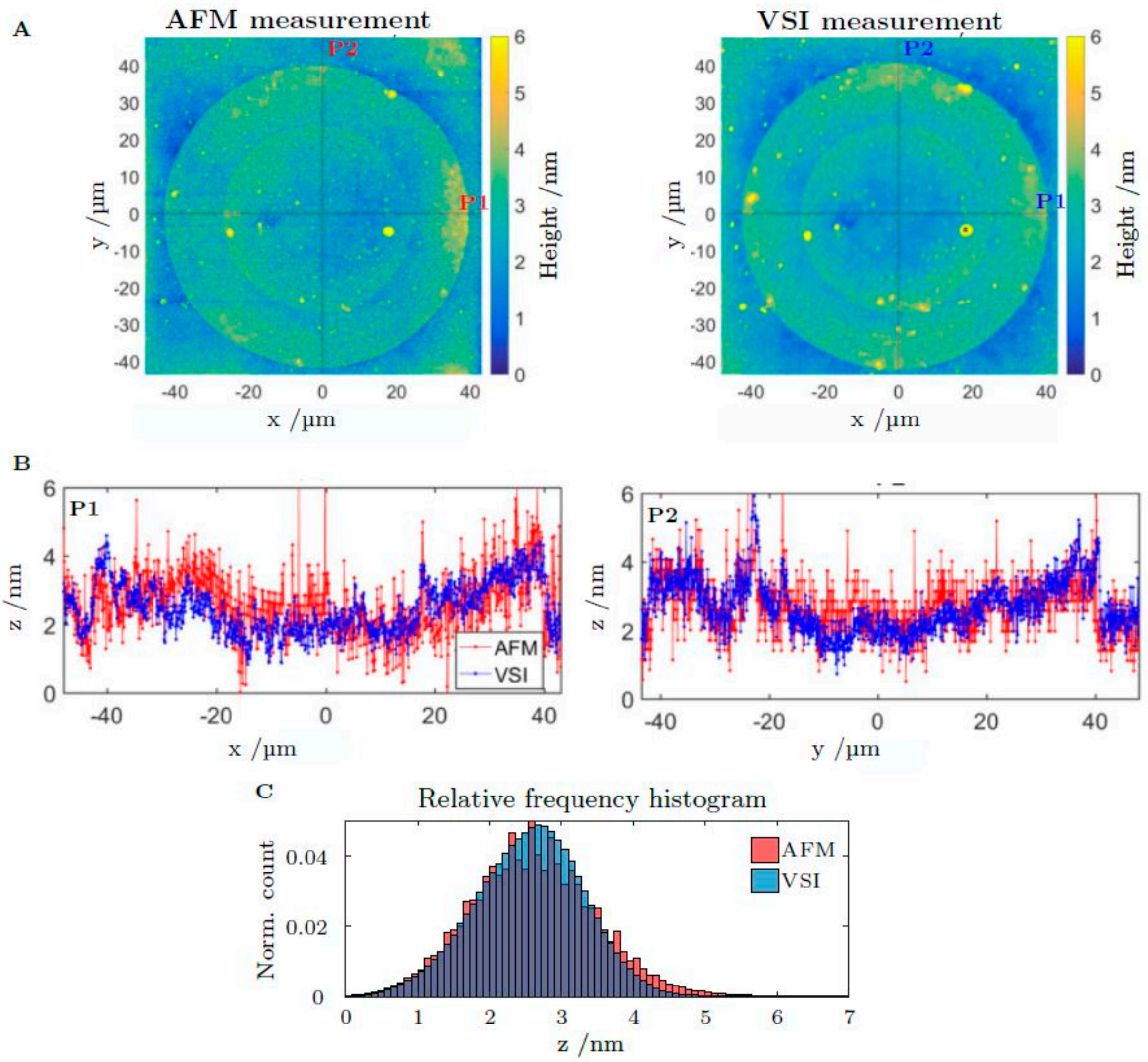

Figure S2. Comparison of VSI measurement to AFM (z-offset adjusted). A left: Topology of a spot measured by AFM (76.1 nmpixel−1 lateral resolution). A software update enabled a new measuring mode: Scan Asyst mode. This mode utilizes peak force tapping but decouples the cantilever response from resonance dynamics to automatically adjust all critical imaging parameters and a real-time feedback loop constantly monitors and adjusts the gain. During scanning, the z-limit is automatically lowered, the set-point is automatically adjusted to minimal forces required and also the scan-rate is automatically controlled. As already seen in figure B.5.2, artifacts appearing as scratches in direction of the cantilever movement are present in the AFM measurement. A right: VSI measurement (74.6 nmpixel−1 lateral resolution at x 50-fold magnification). Red lines indicate measured line profiles depicted in B. Orientation of measurements are rotated 90◦ to the right, compared to 3.3.2 A and B. B: Line profiles for the AFM and VSI measurement. AFM profiles in red, VSI profiles in blue. Vertically: P2. Horizontally: P1. The influence of flattening is more dominant for AFM (obtained data points describe a curve). C: Stacked histograms of AFM and VSI measurements of the spot. The histogram representing the AFM measurement is closer to the VSI one than in B.5.2 due to better matching resolution. However, the AFM histogram is slightly broader and considerably noisier.

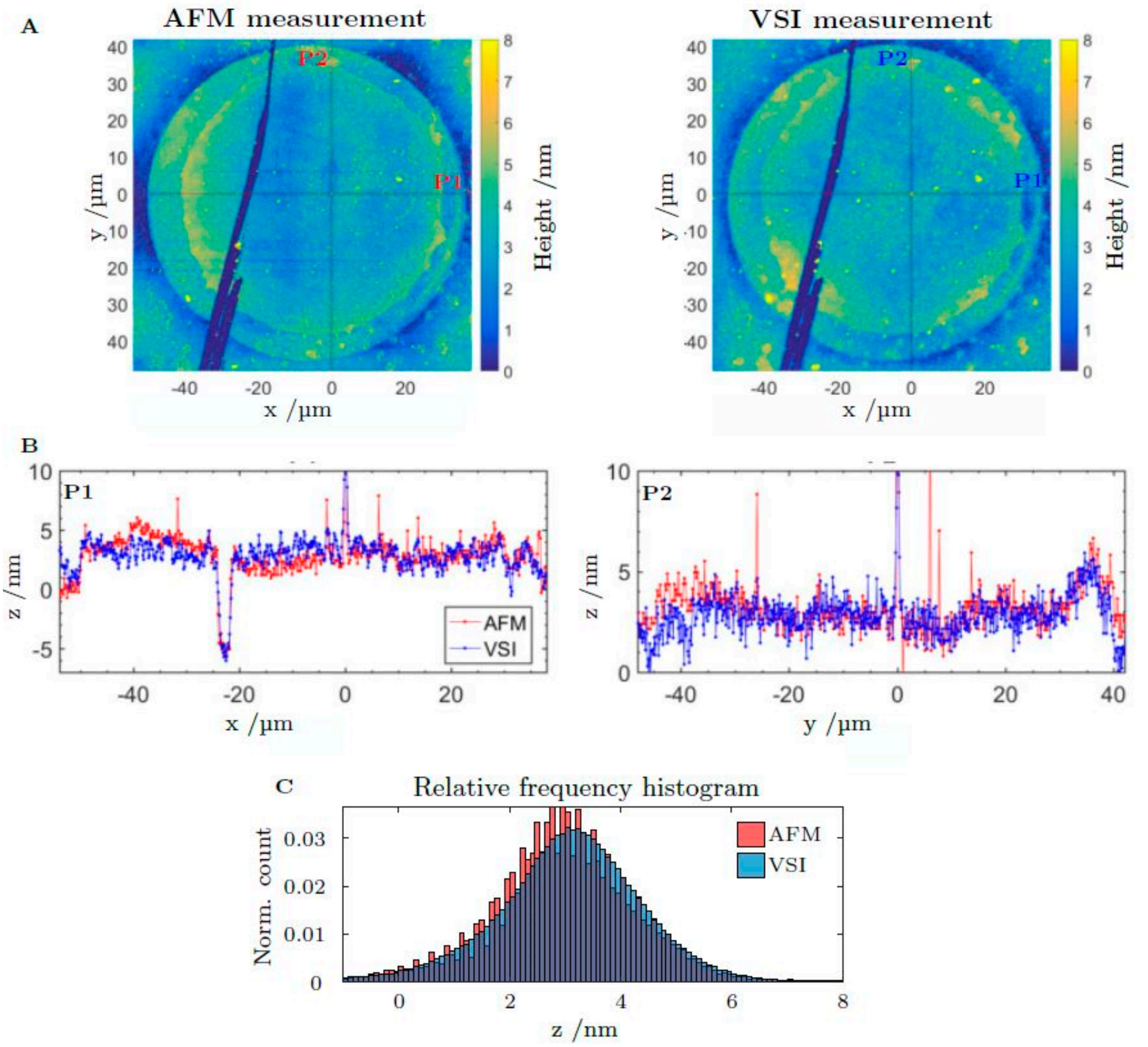

Figure S3. Comparison of VSI measurement to AFM (z-offset adjusted). A left: Topology of SOI measured by AFM lateral resolution is not square: x 181.6 nmpixel−1, y 186 nmpixel−1). Peak force tapping mode was used as measuring mode since the array surface is soft and tends to contaminate the cantilever, which leads to a failing feedback and cantilever consumption. Peak force tapping mode is non-resonant since the oscillation frequency is kept below the cantilever resonance. The mode also allows direct force control and thus minimizes lateral forces which may result in damage of the substrate. The z-position is modulated by a sine wave instead of a triangular one to avoid unwanted turning points. However, artifacts, appearing as scratches in direction of cantilever movement, can be seen in the AFM measurement. A right: Topology of SOI measured by VSI (135.3 nmpixel−1 lateral). VSI data result from one single measurement at 50-fold magnification and was not stitched. Red lines indicate measured line profiles depicted in B. B: Line profiles for the AFM and VSI measurement. AFM profiles in red, VSI profiles in blue. Vertically: P2. Horizontally: P1. C: Stacked histograms of AFM and VSI measurements of the SOI. The higher resolution of the VSI is reflected in the histogram in higher pixel counts. From these measurements it cannot be concluded whether the differences in the line profiles is due to different binning or a result of measurement deviation. Also, the positioning of the profiles is a potential cause of error, since it was done manually and may vary within ± 5 pixel.
